# Supplementary figures and images for: AGER-1 Long Non-Coding RNA Levels Correlate with the Expression of the Advanced Glycosylation End-Product Receptor, a Regulator of the Inflammatory Response in Visceral Adipose Tissue of Women with Obesity and Type 2 Diabetes Mellitus
Source: Int J Mol Sci. 2023 Dec 13;24(24):17447. doi: 10.3390/ijms242417447 (PMC10743952; doi:10.3390/ijms242417447)

Supplementary Figure S1

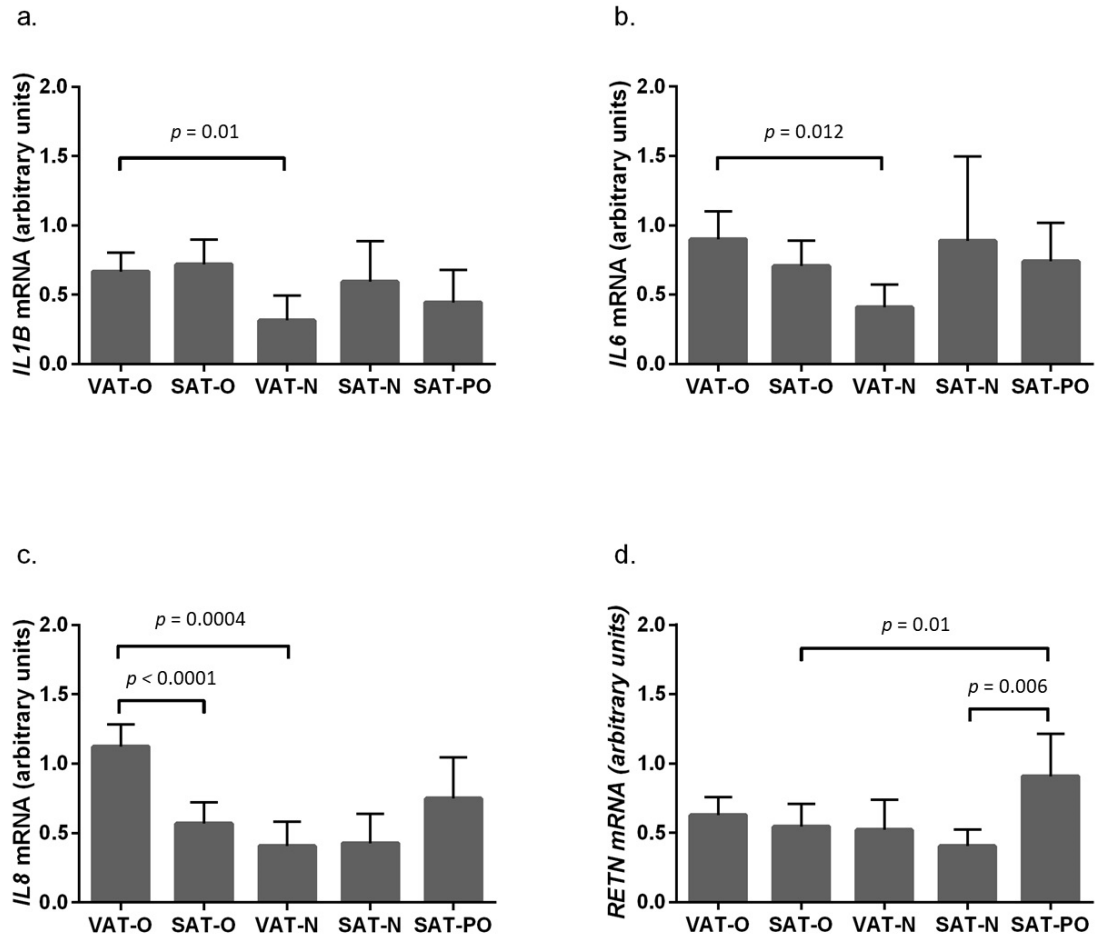

Supplementary Figure S2

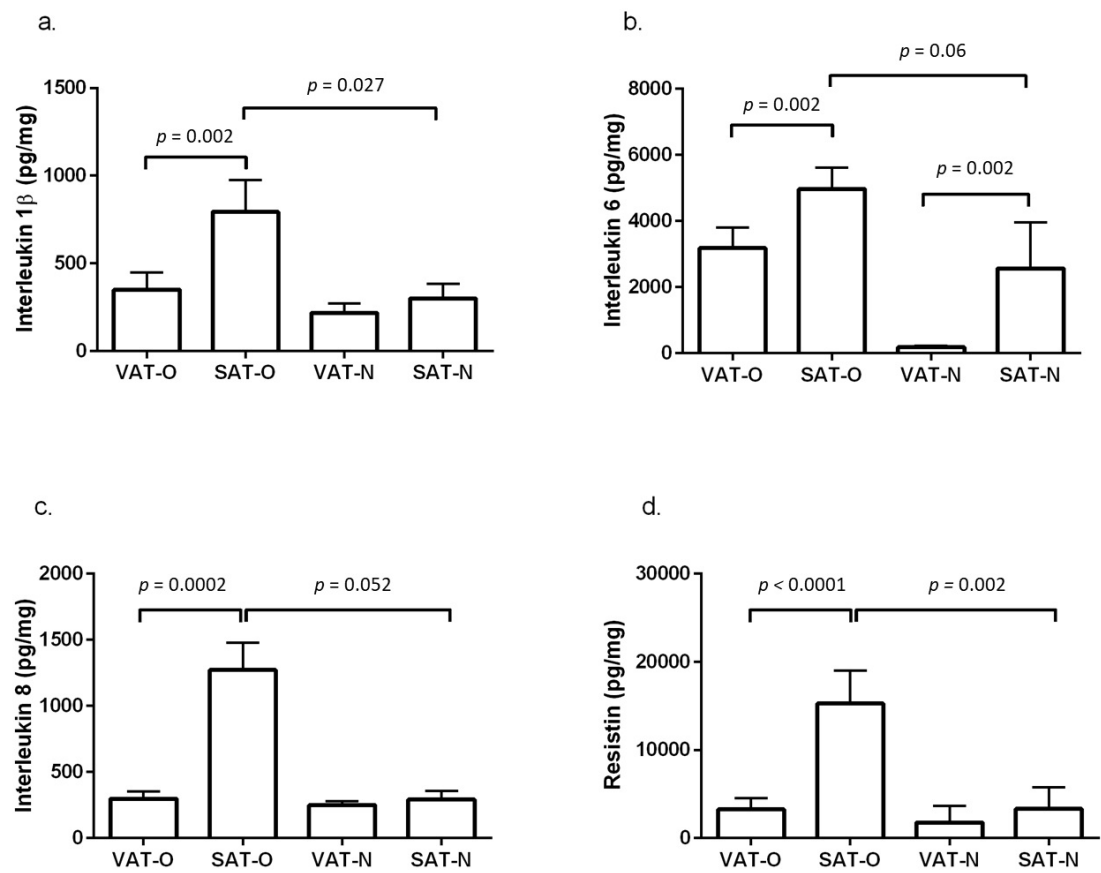

Supplementary Figure S3

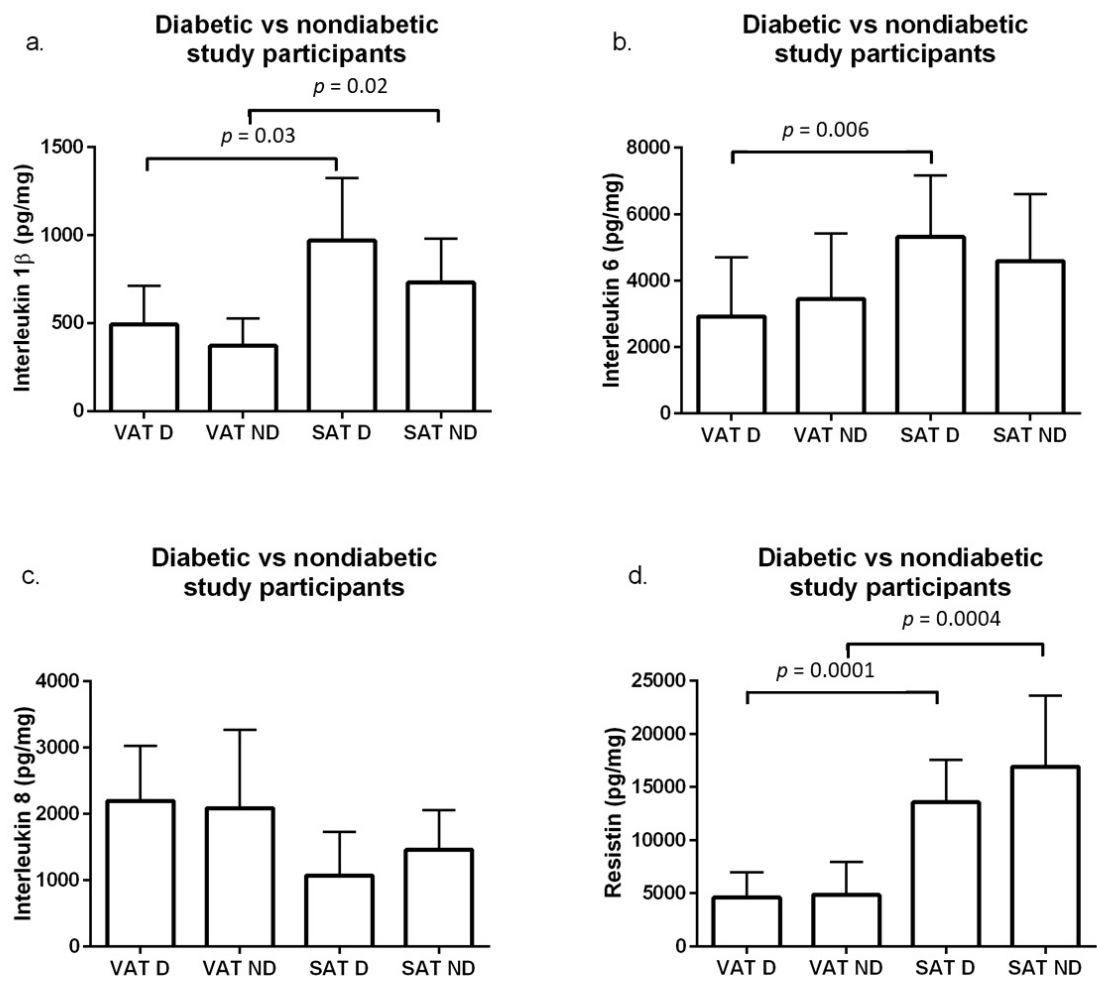

Supplement: Supplementary file 1 [file ijms-24-17447-s001.zip › ijms-2748326-supplementary.pdf]
